# Endometrial Stromal Nodule: Report of a Case

**DOI:** 10.1155/2011/260647

**Published:** 2011-03-07

**Authors:** F. Z. Fdili Alaoui, H. Chaara, H. Bouguern, M. A. Melhouf, H. Fatemi, A. Belmlih, A. Amarti

**Affiliations:** ^1^Department of Obstetrics and Gynecology II, CHU Hassan II, Fez, Morocco; ^2^Department of Anatomic Pathology, CHU Hassan II, Fez, Morocco

## Abstract

Endometrial stromal nodule (ESN) is the least common of the endometrial stromal tumors. They are rare neoplasms which are diagnosed in most instances by light microscopy. Although such nodules are benign, hysterectomy has been considered the treatment of choice to determine the margins of the tumor required for diagnosis and to differentiate it from invasive stromal sarcoma Whose prognosis is totally different. We report a case of a 45 years old woman, with presurgical diagnosis of adnexal mass or uterine tumor. She underwent a total abdominal hysterectomy. Pathologic examination revealed an endometrial stromal nodule. Through this observation, we insist on the fact that the ESNs are rare and benign entities which must be differentiated from the other invasive malignant stromal tumors; this can change the final prognosis.

## 1. Introduction

Tumors of endometrial stroma are very rare mesenchymal tumors of the uterus with cytological and architectural features reminiscent of endometrial stromal cells [1].

The classification of endometrial stromal tumor is difficult and complicated [2, 3]. The recent World Health Organization classification of tumors of the breast and female genital organs divides the uterine stromal neoplasms into three groups: benign endometrial stromal nodule (ESN), low-grade endometrial stromal sarcoma (LGESS), and undifferentiated endometrial sarcoma (UES) [4].

ESN is cytologically similar to low-grade stromal sarcomas, but it is distinguished by its well-circumscribed, expansile margin, and it is considered clinically benign. In contrast, UES is a rare but highly malignant sarcoma lacking overt endometrial stromal differentiation [1].

In this study, we describe a patient with a stromal nodule who underwent a total abdominal hysterectomy, and we insist on the fact that endometrial stromal nodule is a rare disease to be carefully differentiated from other stromal sarcomas, which can change the final prognosis.

## 2. Case

**Figure 1:**
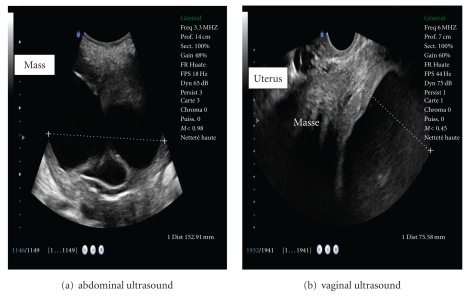
Showed a 15 cm heterogeneous but well-circumscribed mass, consisting of cystic and solid parts, whose relationship with the uterus is not well defined; no vegetation was noted either inside or outside of the mass, and the ovaries were not visualized.

Mrs KR, 45 years old, divorced, G1P1, thyroidectomized under treatment, presented with dull pain and discomfort in the lower abdomen that had lasted for several weeks. The patient had her Menarche at the age of 14, and the menstrual cycles were regular without abnormal uterine bleeding. The abdominal and pelvic examination revealed the abdominal pelvic painless mass reaching the umbilical point, some parts of this mass are soft but the mass dependence on the uterus was not established; the rest of examination was without particularity. Abdominal and vaginal ultrasound showed a 15 cm heterogeneous but a well-circumscribed mass, consisting of cystic and solid parts whose relationship with the uterus is not well defined. No vegetation was noted either inside or outside of the mass. The ovaries was not visualized (Figure 1). Magnetic resonance imaging (MRI) was indicated to specify the seat of the mass and its relationship with the neighborhood organs, this MRI has not been done because of the patient lack of means. The hemoglobin was paradoxically 8,8 g/dl. Laboratory investigations including serum CA 125, T3, T4, TSH were normal. After being transfused for anemia, a laparotomy was done to evaluate the nature of the mass and extent of the disease. With the exception of slight uterine enlargement (14 cm), operative findings were totally normal. The adnexa appeared normal; the patient underwent abdominal hysterectomy. Because of the suspicion of leiomyoma perioperatively and the age of the patient, the tubes and ovaries were preserved. 

**Figure 2:**
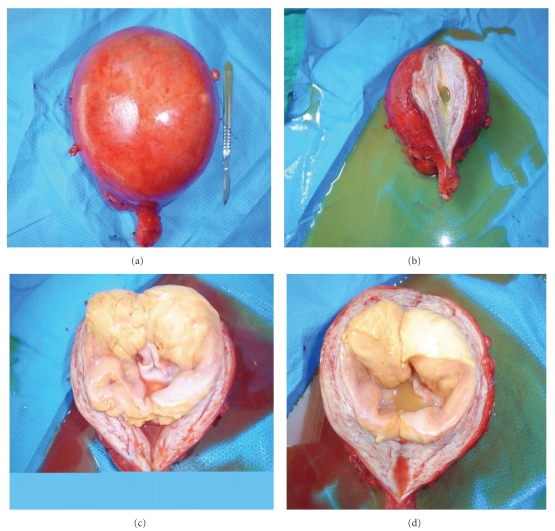
The gross inspection of hysterectomy specimen revealed a well-circumscribed yellow tumor measuring 11/10 cm that appears within the myometrial layer that resembled a leiomyoma.

Gross finding showed a polypoid and protrude tumor involving in the myometrium and tend to bulge above the surrounding myometrium. The tumor has a well-circumscribed contour, measuring 11 × 10 × 8 cm and has a fleshy yellow surface (Figure 2). This tumor is intramural with no connection to the endometrium.

In microscopic findings, the tumor consists of cells that closely resemble normal proliferative-phase endometrial stromal cells with areas of epithelial-like structures that have an appearance reminiscent of an ovarian sex cord-stromal tumor. The tumor cells have uniform, small, darkly staining round or oval nuclei with granular chromatin and inconspicuous nucleoli. Mitotic activity is less than 3MF/10HPF. 

**Figure 3:**
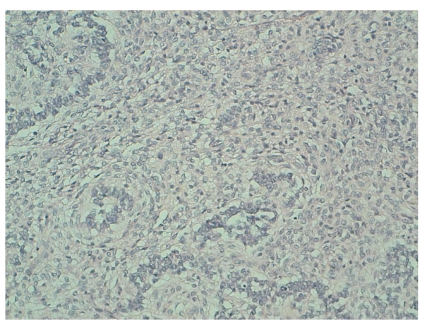
Microscopic finding: standard coloration (HES).

The epithelial-like cells grow in cords and trabeculae, they are cuboidal with scanty amphophilic cytoplasm and nuclei resemble those of the surrounding stromal cells (Figure 3). The tumor presents expansile, noninfiltrative margins that compress the surrounding myometrium. 

**Figure 4:**
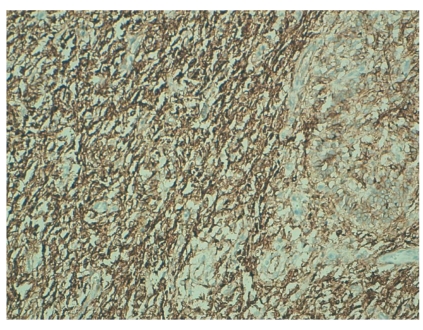
Microscopic finding: immunohistochimy coloration.

The tumor was immunoreactive to CD10 (Figure 4) and hormonal receptors: oestrogen receptor (ER) and progesterone receptor (PR). Immunostaining for AML (alpha smooth muscle), desmine, calretinin, cytokeratin AE1/AE3, and inhibin were negative.

One year after surgery, the woman remains asymptomatic, and is clinically and ultrasonographycally free of disease.

## 3. Discussion

Endometrial stromal tumors are among the least common neoplasms of the uterine corpus, with an annual incidence of about 2 per million women [1–4].

Benign endometrial stromal nodule is a rare subtype that accounts for about one fourth of the endometrial stromal tumors which constitute less than 5% of uterine tumors [5–7].

The diagnosis is in most instances established by light microscopy. The existence of circumscribed benign neoplastic proliferations of endometrial stromal cells, now known as “endometrial stromal nodules” has been known for many years but the literature on these lesions is scanty. Although large series of endometrial stromal tumors often include ESN, they are usually few in number [8] and there is only one large series of cases reported by Tavassoli and Norris in 1981 about 60 cases of ESN [9] and one probable group of 11 cases in the older literature [10]. Both those studies were published before the widespread recognition of the extent to which endometrial stromal tumors, including ESN, could be mimicked by highly cellular leiomyomas. They also antedated the existence of antibodies that facilitate the distinction between EST and smooth muscle tumors and help delineate the endometrial stromal and smooth muscle components in EST with smooth muscle metaplasia. Recently, Dionigi published a series of 50 cases including EST that had an entirely circumscribed margin or had limited focal infiltration at their periphery, and he retained only four endometrial stromal nodules [11]. Also, Amanjit published 5 cases of EST/1261 endometrial neoplasms from January 2001 to December 2004 that correspond to 0,3% of endometrial neoplasms; one case was diagnosed as endometrial stromal nodule [12]. Endometrial stromal nodule has been defined as a well-circumscribed endometrial stromal tumor; however, focal irregularities or finger-like projections into the adjacent myometrium are acceptable if none of them exceed 2 to 3 mm [8, 9].

ESNs like other uterine neoplasms of stromal origin, occur primarily in peri- and postmenopausal women. Tavassoli and Norris [8, 9] reviewed 60 women with endometrial stromal nodules; the median age was 47 years. The clinical presentation is nonspecific; the patients may present with vaginal bleeding, enough to produce anemia, pelvic or abdominal pain or discomfort, or may be asymptomatic [5, 6, 8, 13]. Our patient had a lower abdominal discomfort, without menorrhagia but paradoxically the hemoglobin was 8,8 g/dl (hiding an anemia) which can be explained as underestimated anemia or menorrhagia. The most common preoperative diagnoses were leiomyoma and adnexal masses [1, 13].

Because the majority of patients are beyond childbearing years, a hysterectomy is usually required; it permits the thorough evaluation of the tumor margin too, which is necessary to distinguish a benign stromal nodule from a stromal sarcoma [3, 14]. However, in contrast to stromal sarcomas, patients with stromal nodules have remained free of disease and no recurrences were noted following hysterectomy [9, 13].

In a curettage specimen, distinction between ESN and low-grade ESS is almost impossible, unless the tumor is very small and the margins can be fully evaluated. In women of reproductive age who desire to preserve fertility, diagnostic imaging and hysteroscopy may be used to follow up tumor growth. In some cases, hormonal therapy with local excision may be successful. In the series reported by Tavassoli and Norris, six patients underwent simple excision of the uterine nodules. One patient had a hysterectomy 4 years later for endometrial hyperplasia, and the pathologic evaluation revealed no evidence of stromal tumor. The other five patients were followed from 6, 2 to 10 years with no evidence of recurrence [9]. Schilder[13] published a successful hormonal therapy (leuprolide acetate) in decreasing the size of a low-grade endometrial stromal sarcoma, the patient underwent local excision of the tumor with preservation of reproductive function. Although the receptor status of stromal nodules has not been studied, their similarity on a cellular level to low-grade stromal sarcomas suggests that a trial of hormonal therapy in this case, when conservative management was desired, might be successful. The decrease in tumor size permitted local excision and preserved reproductive function [13].

Our patient aged 45 years was avid neither for pregnancy nor for a conservative treatment.

Macroscopically, the tumor is characteristically a solitary, well-delineated, round fleshy nodule with a yellow to tan sectioned surface. The median tumour diameter is 4 cm (range 0.8 to 15 cm). It was 11 cm in our case. About two thirds are purely intramural without any apparent connections to the endometrium. Occasional tumors are cystic, but foci of necrosis and hemorrhage are rare.

Differential diagnosis of an endometrial stromal nodule depends on microscopic findings.

The histological appearance found endometrial stromal nodules with areas of epithelial-like structures that resemble ovarian sex cord tumors. The stromal nodules have expansile, noninfiltrative margins that compress the surrounding endometrium and myometrium. Minor irregularities of the margin are common, but invasion of the surrounding myometrium indicates that the tumor is a stromal sarcoma, not a stromal nodule [11, 15].

Endometrial stromal tumors with sex cord-like elements exhibit a polyphenotypic immunophenotype. There is most often a mixed epithelial-myoid phenotype, with immunoreactivity for cytokeratin and actin, and, in some cases, desmin [16]. Immune-stains for EMA are almost always negative. In accord with the resemblance to a sex cord tumor, immunoreactivity for inhibin and CD99 is detected in epithelial-like structures in about a third of this type of tumors. 

In our case, the tumor expresses the CD10 and hormonal receptors. Immunostaining for AML (alpha smooth muscle), desmine, calretinin, cytokeratin AE1/AE3, and inhibin were negative.

Endometrial stromal nodule with focal sex cord-like differentiation tend to relapse and metastase. In Clement and Scully initial report, three of five patients with followup had recurrences and two died [16].

## 4. Conclusion

We present a patient with endometrial stromal nodule. 

There is no reliable preoperative diagnostic procedure to identify this tumour. Clinical presentation is nonspecific. Hysterectomy is the treatment of choice. 

The diagnosis is done on microscopic examination. The margins of tumour must be determinate to differentiate it from invasive stromal tumors.

Considered as benign tumor, the prognosis is excellent when the diagnosis is sure. 

## References

[1] Elagoz S, Kıvanc F, Aker H (2005). Endometrial stromal tumors—a report of 5 cases. *Aegean Pathology Journal*.

[2] Ramirez NC, Lawrence WD (2003). Endometrial stromal lesions. *Weitner Cote Suster Weiss. Modern Surgical Pathology*.

[3] Zaloudek C, Hendrickson MR, Kurman RJ (2001). Mesenchymal tumors of the uterus. *Blaustein’s Pathology of the Female Genital Tract*.

[4] Tavassoli FA, Devilee P (2003). *World Health Organization Classification of Tumors. Pathology and Genetics of the Breast and Female Genital Organs*.

[5] Ryuko K, Takahashi K, Miyazaki K (1996). A case of endometrial stromal nodule. *Shimane Journal of Medical Science*.

[6] Silverberg SG, Kurman RJ (1992). *Tumors of the Uterine Corpus and Gestational Trophoblastic Disease*.

[7] Hoskins WJ, Perez CA, Young RC (1992). *Principles and Practice of Gynecologic Oncology*.

[8] Kim KR, Jun SY, Park IA, Ro JY, Nam JH (2005). Endometrial stromal tumor with limited infiltration and probable extrauterine metastasis: report of a case. *Annals of Diagnostic Pathology*.

[9] Tavassoli FA, Norris HJ (1981). Mesenchymal tumors of the uterus. VII. A clinicopathological study of 60 endometrial stromal nodules. *Histopathology*.

[10] Michaeles L, Langley FA (1957). Mesenchymal tumours of the uterus resembling the haemangiopericytoma. *The Journal of Obstetrics and Gynaecology of the British Empire*.

[11] Dionigi A, Oliva E, Clement PB, Young RH (2002). Endometrial stromal nodules and endometrial stromal tumors with limited infiltration: a clinicopathologic study of 50 cases. *American Journal of Surgical Pathology*.

[12] Bal A, Mohan H, Aulakh R, Huria A (2008). Endometrial stromal lesions: a morphological and immunohistochemical study of short series. *Archives of Gynecology and Obstetrics*.

[13] Schilder JM, Hurd WW, Roth LM, Sutton GP (1999). Hormonal treatment of an endometrial stromal nodule followed by local excision. *Obstetrics & Gynecology*.

[14] Yilmaz A, Rush DS, Soslow RA (2002). Endometrial stromal sarcomas with unusual histologic features: a report of 24 primary and metastatic tumors emphasizing fibroblastic and smooth muscle differentiation. *American Journal of Surgical Pathology*.

[15] Chang KL, Crabtree GS, Lim-Tan SK, Kempson RL, Hendrickson MR (1990). Primary uterine endometrial stromal neoplasms. A clinicopathologic study of 117 cases. *American Journal of Surgical Pathology*.

[16] Geetha V, Rupashree S, Bhat S (2008). Endometrial stromal nodule with smooth muscle differentiation. *Indian Journal of Pathology & Microbiology*.

